# Surgical Outcomes of Total Anomalous Pulmonary Venous Connection Repair

**DOI:** 10.3390/medicina58050687

**Published:** 2022-05-23

**Authors:** Radoslaw Jaworski, Andrzej Kansy, Joanna Friedman-Gruszczynska, Katarzyna Bieganowska, Malgorzata Mirkowicz-Malek

**Affiliations:** 1 Department of Anesthesiology and Intensive Care, Faculty of Medicine, Medical University of Gdansk, 80-214 Gdansk, Poland; 2 Department of Cardiothoracic Surgery, The Children’s Memorial Health Institute, 04-736 Warsaw, Poland; ankansy@gmail.com (A.K.); joanna@friedman.pl (J.F.-G.); mmal10@poczta.onet.pl (M.M.-M.); 3 Department of Cardiology, The Children’s Memorial Health Institute, 04-736 Warsaw, Poland; kbieganowska@wp.pl

**Keywords:** TAPVC, total anomalous pulmonary venous connection, pediatric cardiac surgery, congenital heart disease

## Abstract

*Background and Objectives:* Over the years, surgical repair of total anomalous pulmonary venous connection (TAPVC) outcomes have improved, however, morbidity and mortality still remain significant. This study aims to assess the early and long-term outcomes of surgical treatment of TAPVC children, operated on between 2006 and 2016, in one pediatric center in Poland. *Materials and Methods*: Diagnostics, surgical treatment, and follow-up data from 83 patients were collected. In addition, survival and risk factor analyses, control echocardiographic, and electrocardiographic examinations were performed. *Results*: In the analyzed group (*n* = 83), there were seven hospital deaths (within 30 days after the operation) (8.4%) and nine late deaths (10.8%). The mean follow-up time was 5.5 years, and, for patients who survived, it was 7.1 years. The mean survival time in patients with completed follow-up (*n* = 70) was 10.3 years; the overall five-year survival rate was 78.4%. Independent mortality risk factors were type I TAPVC, single ventricle physiology, time from admission to operation, intensive care unit stay, postoperative hospital stay, and temporary kidney insufficiency requiring dialysis. *Conclusions*: The presence of single ventricle physiology and the supracardiac subtype of TAPVC might be negative prognostic factors, while normal heart physiology presents with good post-repair results. This study indicates that cardiac arrhythmias may occur. Morbidity and mortality, related to surgical TAPVC correction, still remain significant.

## 1. Introduction

Total anomalous pulmonary venous connection (TAPVC) is a rare congenital heart disease (CHD), occurring in neonates and accounting for about 3% of all CHD [1,2]. In this lesion, the pulmonary veins commonly connect to a venous confluence that, ultimately, drains to the right atrium instead of to the left atrium. The most commonly used anatomical classification of TAPVC, so-called modified Darling classification, was proposed by Craig, Darling, and Rothney [3]. Based on the drainage pattern of the pulmonary venous return to the systemic venous circulation, four types of TAPVC were described. In supracardiac TAPVC (type I), all pulmonary veins enter superior vena cava via an ascending vertical vein. Cardiac TAPVC (type II) occurs when the pulmonary veins connect to the right atrium via the coronary sinus or directly into the right atrium. Infracardiac TAPVC (type III) occurs when the common confluence connects to either the portal vein or the inferior vena cava. Finally, the mixed type of TAPVC (type IV) consists of a variable number of connections that drain directly to the heart or to an additional extracardiac structures. Surgical repair remains the only therapeutical option; however, it is related to significant morbidity and mortality. Historically, TAPVC was related to the mortality rate, as high as 80% in the first year, without any surgical intervention [1]. Over the years, with advances in surgical techniques, diagnostic accuracy, and perioperative management, the outcomes of surgical treatment have improved; however, the main problem is early mortality as well as postoperative pulmonary venous occlusion (PVO) [4]. Obstructed venous return can be expected in roughly one-third of TAPVC cases, and the outcome is favorable in less than half of the patients [5]. TAPVC in children with univentricular heart physiology or heterotaxy syndrome remains a therapeutical challenge [5,6].

This study aims to assess the early and long-term outcomes of surgical treatment of TAPVC patients, in one pediatric center in Poland. Additionally, we tried to determine predictive factors for morbidity and mortality.

## 2. Materials and Methods

### 2.1. Study Design

**Figure 1:**
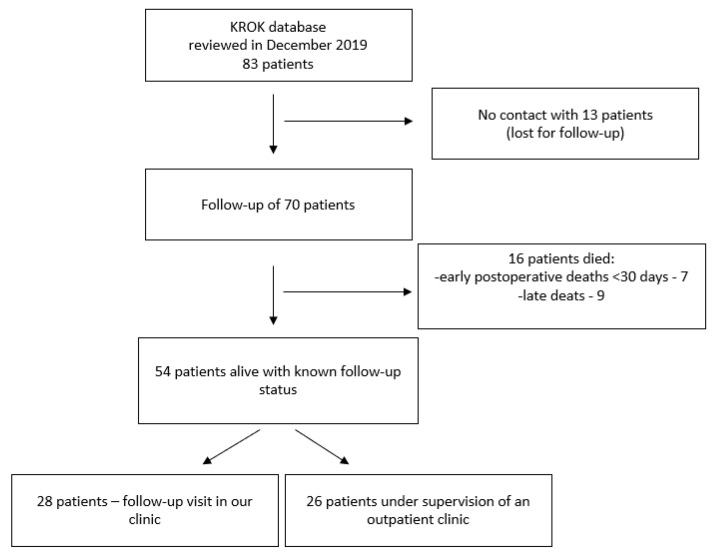
Study scheme.

This study was conducted in the Department of Cardiothoracic Surgery, Children’s Memorial Health Institute in Warsaw, Poland. Pediatric patient data from the Polish National Registry of Cardiac Surgery Procedures (KROK) database, with TAPVC diagnosis who were admitted to the clinic from 2006 to 2016, were reviewed [7]. Diagnostics and surgical treatment data of 83 patients from the medical history were collected in December 2019, although 16 patients from this group died. We established communication with the parents of the patients. In total, 28 of them agreed to come to our clinic for a follow-up visit, while the other 26 patients were under the supervision of the outpatient clinic but did not want to come to our clinic for an additional follow-up study. Unfortunately, in 13 cases (15.7%), we could not communicate with the patients and parents after hospital discharge and surgical treatment, so they were lost to follow-up. The study design is presented in Figure 1. The study was approved by the Children’s Memorial Health Institute Ethics Board (18/KBE/2018) on 27 June 2018. It was performed following the relevant guidelines and regulations and was financed by our hospital’s internal sources (internal grant No. S184/19). Patient consent was obtained during a follow-up examination in 28 patients and waived in other patients, due to only retrospective assessment.

### 2.2. Patient Cohort

A total of 83 patients, who underwent TAPVC surgical correction, were evaluated. There were 57 male (68.7%) and 26 female patients (31.3%). The mean age at operation was 13 days (SD = 43.9; min. = 1; max. 205), with mean body weight at the operation of 3.4 kg (SD = 0.9; min. = 1.7; max. = 6.7). Ten children (12.5%) weighed below 2.5 kg. Anatomical TAPVC types were described according to the modified Darling’s classification [3]; there was supracardiac TAPVC (type I) in 37 children (44.6%), intracardiac TAPVC (type II) in 14 patients (16.9%), infracardiac TAPVC (type III) in 28 children (33.7%), and mixed TAPVC (type IV) in 4 patients (4.8%). Preoperatively restrictive TAPVC was defined as a restriction in the pulmonary venous outflow observed in echocardiography, need of operation in the first 30 days of the child’s life, or need of operation within 3 days after hospital admission. Such a defined preoperative restriction was confirmed in 69 (83.1%) patients. Detailed patient characteristics are presented in Table 1.

### 2.3. Surgical Data

The TAPVC repair was performed via median sternotomy in all patients, and a classic approach with deep hypothermic circulatory arrest (DHCA) was performed in 73 patients (88%). Cardiopulmonary bypass (CPB) time was defined as the time from CPB start to CPB stop, together with DHCA time. Surgical and postoperative data are presented in Table 2. Postoperative complications, including mechanical ventilation over seven days and renal insufficiency requiring dialysis, arrhythmia, and infections, were evaluated.

Survival analysis, with risk factor analysis for mortality, was performed in 70 patients, with a known follow-up status of at least survival. Risk factors in univariate and multivariate analyses were performed. In addition, the long-term status of 54 alive patients, with a known follow-up status, was evaluated, according to the need for reoperation due to PVO; arrhythmias (requiring permanent heart stimulation or not) were assessed in a 24-h ECG, and PVO signs were evaluated during echocardiography. The end date of the follow-up status was 8 January 2020.

### 2.4. Statistical Analysis

The mean value, standard deviation, and range were evaluated for continuous variables. Categorical variables were described in terms of the number and percentage of each subgroup; the respective values were rounded up to one decimal place. The median value with standard deviation and the range were evaluated for continuous variables. Data that did not follow a normal distribution were analyzed, using the nonparametric Mann–Whitney U test. The relationships between categorical variables were assessed, using Pearson’s chi-squared test, and, in the case of subgroups comprising less than five cases, Yates’ correction was applied. A value of *p* < 0.05 was chosen as the cut-off point for significance.

Survival analysis was performed using the Kaplan–Meier estimation, and comparisons between subgroups were made using the log-rank test. Risk factors in univariate and multivariate analysis were tested, using the Cox proportional hazards model. Variables with *p* ≤ 0.2 in univariate analysis were included in the multivariate analysis.

Statistical analysis was carried out using SPSS version 20.0 (SPSS Inc., Chicago, IL, USA).

## 3. Results

**Figure 2:**
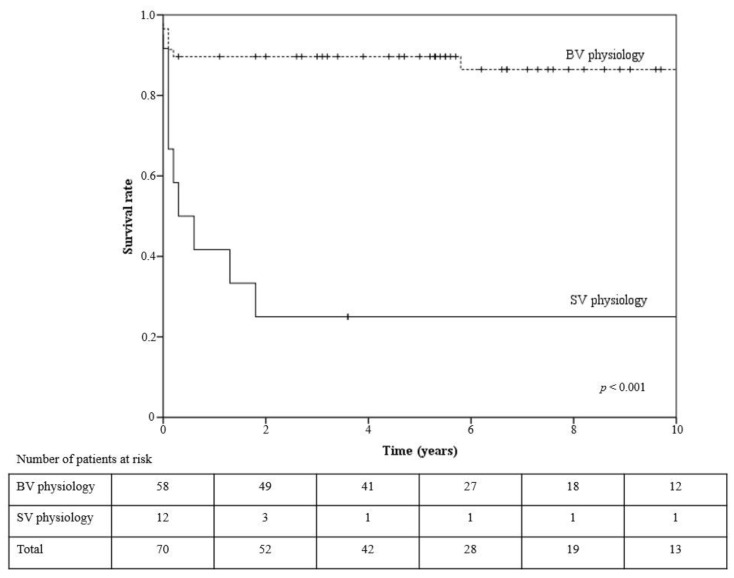
Kaplan–Meier survival curves for biventricle vs. single ventricle physiology patients after TAPVC correction (*p* < 0.001). + -—observation censored, SV—single ventricle, BV—biventricle.

In the analyzed group (*n* = 83), there were seven early deaths (within 30 days after the operation) (8.4%) and nine late deaths (10.8%). The mean follow-up time was 5.5 years (SD = 4.2; min. = 0; max. = 13.6), and for patients who survived, it was 7.1 years (SD = 3.5, min.= 0.3; max. = 13.6). The mean survival time in patients with completed follow-up (*n* = 70) was 10.3 years (SD = 0.67; 95%CI:9–11.6); the five-year survival rate was 78.4%, i.e., 89.7% in the normal-heart-physiology group and 25% in the single-heart-physiology group (*p* < 0.001) (Figure 2). The Kaplan–Meier survival analysis showed that in the observed group, only the complications defined in the methods (*p* < 0.001), as well as separately, for mechanical ventilation over seven days (*p* < 0.001), temporary kidney insufficiency requiring dialysis (*p* < 0.001), and postoperative arrhythmia (*p* = 0.022), were associated with increased mortality. No differences were observed for gender, restriction, TAPVC type, weight (<3 kg), newborns, DHCA usage, delayed sternum closure, and major infections. In patients with BV physiology, better survival was related to other-than-type I TAPVC (*p* = 0.017), no complications (*p* = 0.003), ventilation below seven days (*p* = 0.001), no need of dialysis (*p* < 0.001), and no need for reoperation (*p* = 0.015).

Univariate and multivariate mortality analyses were performed for preoperative, intraoperative, and postoperative factors, as shown in Table 3. Significant variables associated with mortality, in a univariate Cox analysis, were entered into a multivariate analysis. In the analysis of the preoperative factors, children with type 1 TAPVC (HR = 2.568; *p* = 0.068), SV physiology (HR = 9.125; *p* < 0.001), and weight below 3 kg (HR = 2.242; *p* = 0.112) had poorer outcomes, however, in multivariate analysis, this was confirmed only for type I TAPVC (HR = 3.61; *p* = 0.017) and SV physiology (HR = 9.126; *p* < 0.001). Based on univariate analysis of intra- and postoperative factors, time from admission to operation (HR = 1.015; *p* = 0.114), CBP time (HR = 1.007; *p* = 0.013), postoperative mechanical ventilation time (HR = 1.001; *p* = 0.001), ICU stay (HR = 1.013; *p* = 0.001), postoperative hospital stay (HR = 1.014; *p* = 0.001), postoperative complications (HR = 63.4; *p* = 0.044), postoperative mechanical ventilation over seven days (HR = 73.552; *p* = 0.037), temporary kidney insufficiency requiring dialysis (HR = 34.7; *p* = 0.001), postoperative arrhythmia (HR = 1.538; *p* = 0.047), and need for reoperation (HR = 2.944; *p* = 0.154) were factors included in the multivariate analysis. In the multivariate analysis, a significant influence on survival was confirmed for the time from admission to operation (HR = 1.657; *p* < 0.001), ICU stay (HR = 1.624; *p* < 0.001), postoperative hospital stay (HR = 0.608; *p* < 0.001), and temporary kidney insufficiency requiring dialysis (HR = 38.4; *p* = 0.005).

Forty follow-up patients (74.1%) had a 24-h ECG evaluation, with a mean time from TAPVC correction to examination of 7.8 years (SD = 3.1; range: 2.9–13.1 years). The mean observed heart rate was 87 beats per min. (SD = 14.8; range: 52–145 per min.), with a mean minimal heart rate of 52 beats per min. (SD = 11.1; range: 33–91 per min.) and a mean maximal heart rate of 161 beats per min. (SD = 23; range: 105–209 per min.). During 24-h ambulatory ECG monitoring, 29 children remained in sinus rhythm; in 3, junctional escape beats were recorded, and 1 patient had long periods of sinus tachycardia. In nine patients, sinus rhythm coexisted with a different atrial pacemaker rhythm, and one also had junctional escape beats. In one boy, the persistent atrial ectopic rhythm was registered. In addition, one girl had a pacemaker implanted in the early postoperative period, due to sinus node dysfunction, and sinus rhythm alternated with AAI pacing. In 19 (47.5%) of the 24-h ECG monitored patients, cardiac arrhythmias were recorded. In 12 patients, some supraventricular premature contractions were found (<0.5% of all beats); in 1 boy, over nine years after surgery, supraventricular arrhythmia accounted for 2.3% of all beats (there were single contractions, 13 pairs, and 12 episodes of supraventricular tachycardia lasted up to 20 s), while 4 children had single premature ventricular beats (which constituted <1% of all beats). In two children, periodic prolongation of the PR interval was recorded as a first-degree atrioventricular block, and one of these children had a transient complete atrioventricular block at the early post-operation time.

Follow-up echocardiography evaluation of 28 patients demonstrated mean blood flow, in pulmonary venous to the left atrium anastomosis, of 1.38 m/s (range: 0.6–2.1 m/s), with an accelerated velocity >1.6 m/s in 9 patients (32%). Moreover, a maximal velocity of tricuspid regurgitation jet was evaluated to estimate pulmonary artery pressure—it exceeded the normal value of 2.5 m/s in seven (25%) patients (range: 1.8–2.9 m/s).

Reoperations were performed in four patients (5.7%) in the analyzed group, and two of them died in further follow-up, due to persistent pulmonary hypertension.

## 4. Discussion

In this study, we reviewed the results of 83 children who underwent TAPVC surgical correction at our department, from 2006 to 2016, and the long-term outcomes during a follow-up examination. Based on KROK database analysis, to the best of our knowledge this is the largest TAPVC patient series in Poland. Our patients’ overall operative mortality rate within 30 days after TAPVC corrective surgery was 8.4%, and late mortality was 10.8%; the five-year survival rate in the biventricular and single-heart physiology groups was 89.7% and 25%, respectively. These results, generally, correspond with other single-institution retrospective studies, with operative mortality between 2.7% and 21% and a late mortality of 9.7–24.3% [8,9,10,11,12]. However, the percentage of early deaths significantly decreased over the last years [9,12]. This is thought to be related to the early diagnosis, aggressive treatment of pulmonary hypertensive crisis with the utilization of NO to control pulmonary hypertension pre- and postoperatively, improved surgical techniques, increased surgical expertise, and, most importantly, improved postoperative care [13]. However, the mortality of TAPVC patients, when associated with major cardiac anomalies, including SV heart physiology, remains as high as 41–52%, with a five-year survival rate, for patients with heterotaxy, of 79% [4,13,14,15,16,17]. In patients with SV heart physiology, Sugano et al. report a 5-year survival rate of 58%, and Nakayama et al. report a 10-year survival rate of 51% [6,18]. Padalino et al. report that SV heart physiology is significantly associated with increased operative mortality, with a hazard ratio of 8.7, similar to our results [4]. A sutureless repair could be a reasonable option in this group of patients, however, in our center, we use this technique only in reoperations [19].

Risk factor analysis for mortality in our series showed that preoperative SV physiology and the supracardiac subtype of TAPVC were the main contributors to poorer outcomes, whereas, operatively and postoperatively, those included a longer time from admission to operation, a longer stay in the ICU, a shorter postoperative hospital stay, and postoperative renal insufficiency requiring temporary dialysis. These findings suggest that, apart from the heart disease complexity, postoperative heart failure manifested by postoperative renal insufficiency, requiring temporary dialysis, is the most important risk factor in this children group. In TAPVC patients, the overall increased complexity of perioperative management is fundamental [8]. Different studies show that other risk factors, for mortality in children with TAPVC, are preoperative pulmonary hypertension, acidosis, younger age at operation, body weight <2 kg, other cardiac anomalies, longer ECC time, AoX time over 60 min, postoperative PVO, and posterior anastomosis technique; however, in our series, we did not confirm these observations [4,9,10,11,12]. Probably due to the relatively small number of patients, we did not observe younger age and low body weight as risk factors in multivariate analysis. However, this could correspond in this patient group to the additional emergency operations associated with higher mortality and morbidity, as suggested by other authors [20,21]. The impact of proper preoperative stabilization and control of pulmonary hypertension (introduction of pulmonary vasodilators also in preoperative therapy) is underlined in several studies [4]. However, a longer time from admission to operation seems to be related to poorer outcomes in our series.

We observed that the supracardiac subtype of TAPVC was related to worse outcomes than the other types of TAPVC. However, other authors observed a poor prognosis related, more likely, to the mixed or infracardiac subtypes of TAPVC [4,20,21]. What is worth underlining is that the mixed subtype could be a challenge because of the unpredictability of pulmonary venous connections and the requirement for a combination of techniques, including the individualized anastomosis of pulmonary veins. However, as reported by Xiang et al., the results of the mixed type of TAPVC patient corrections are good, with a hospital mortality rate of 7.7% and a five-year survival rate of 91% [22].

For the needs of our study, we defined preoperatively restrictive TAPVC as a restriction observed in pulmonary venous outflow in echocardiography, the need for operation in the first 30 days of child’s life, or the need for operation within three days after hospital admission. Such defined preoperative restriction was confirmed in 69 (83.1%) patients; however, this did not influence the early mortality in our patients. Four of our patients required reoperations for recurrent PVO (5.7%), and two of them did not survive because of persisting pulmonary hypertension. Other studies show that the need for reoperation due to recurrent PVO ranges between 4% and 17.5% [4,9,10,12,20,23]. Harada et al. show that postoperative PVO diagnosed at <6 months was related to a higher mortality rate than in the other group (64% and 11.1%, respectively) [10]. This is why it is suggested that preventing postoperative PVO could decrease mortality rates in this patient group [10]. More and more papers describe the sutureless technique (i.e., anastomosing the left atrium to the posterior pericardium, rather than to the pulmonary vein tissue itself) for primary TAPVC corrections, with promising early results. However, long-term results are not fully known, yet [12,20,24]. Another unanswered issue concerning the TAPVC correction technique is whether or not the vertical vein should be ligated at the time of TAPVC correction [13]. Based on our data, we could not discuss this subject because we had always ligated the vertical vein. However, Karaci et al. could not demonstrate any statistically significant difference between patients with a closed or opened vertical vein [13]. Zhao et al. conclude that it is beneficial to leave the vertical vein open in patients, when the left atrial pressure is higher than 15 mm Hg [25].

Assessment of the heart rhythm after surgery, for congenital heart defects, is an essential diagnostic element in the follow-up period. Few reports describe cardiac rhythm in patients after TAPVC operations, in which small groups of patients are presented, but arrhythmia is found in almost half of them [26,27,28]. Most often, it was supraventricular arrhythmia with a tendency to bradycardia, including sinus bradycardia, ectopic atrial tachycardia, premature atrial contraction, supraventricular tachycardia, and, less frequently, atrial fibrillation. Out of our 40 patients monitored with a 24-h ECG, such an arrhythmia was found only in 13 children, who did not require therapy. In some children, premature ventricular beats are recorded—in our group, there were four such cases. Atrioventricular conduction disturbances were registered, sporadically—in two of our patients. Permanent cardiac pacing was necessary in individual cases, namely in one girl in our group. Unfortunately, isolated death cases due to complex ventricular arrhythmia and bradycardia were reported [25]. There were no arrhythmic deaths among our patients.

The main outcome of the echocardiographic follow-up was the presence of the diagnostic signs of pulmonary vein obstruction and pulmonary hypertension. However, in the literature, the definition of PVO is not precise. Some of the authors define PVO as a PV flow velocity accelerated >2.5 m/s [12], >1.6 m/s [29,30], while some define it as >1.1 m/s [31]. On the other hand, in normal healthy children, we do not observe PV flow >1 m/s [32]. In our study, we assumed PVO as PV flow >1.6 m/s and found it in 32% of patients. Another echocardiographic outcome of our study was the presence of echocardiographic signs suggesting pulmonary hypertension, defined as a tricuspid regurgitation flow velocity over 2.5 m/s. This was found in a quarter of our patients; however, the augmentation of flow velocity was mild (maximum 2.9 m/s), and it was not correlated with the PVO signs. However, it is worth noting that all of our patients remained in the I NYHA class and were free from cardiac medication.

Our study has several limitations. The major limitation was that the study concerns patients treated in one center only. Moreover, the number of patients available in our study was not a large group; thus, the small number of cases could affect the results. In addition, the echocardiographic assessment, due to the long period of observation, was not accessible in all cases.

## 5. Conclusions

The presence of SV heart physiology and the supracardiac subtype of TAPVC might be negative prognostic factors, for children’s survival after TAPVC correction. When the BV heart physiology of TAPVC presents with good post-repair results, in terms of morbidity and mortality, there still is postoperative PVO, with low incidence, but it remains a serious problem in this children group and requires careful follow up. There is an ongoing discussion about how to prevent postoperative PVO, and it may be that the only strategy to avoid this complication remains to perform an anastomosis, without touching pulmonary veins directly. The study indicates that cardiac arrhythmias can occur in nearly half of otherwise asymptomatic patients, after correction for total anomalous pulmonary venous connection. Thus, these patients require an assessment of the heart rhythm at long-term follow-up, even if they are asymptomatic.

## Figures and Tables

**Table 1:** Patient characteristics referred to TAPVC correction.

Feature	Total (*n* = 83); Mean (SD; Range) or Number (%)	Normal Heart Physiology (*n* = 67); Mean (SD; Range) or Number (%)	Single Ventricle Physiology (*n* = 16); Mean (SD; Range) or Number (%)	*p*-Value
Gender (male)	57 (68.7%)	45 (67.2%)	12 (75%)	0.388
Age at operation (days)	13 (43.9; 1–205)	15 (47.5; 1–205)	10.5 (19.3; 3–63)	0.492
Weight (kg)	3.4 (0.9; 1.7–6.7)	3.5 (0.9; 1.8–6.7)	3 (0.6; 1.7–4)	0.039
TAPVC subtype:				
- supracardiac (I) - intracardiac (II) - infracardiac (III) - mixed (IV)	37 (44.6%) 14 (16.9%) 28 (33.7%) 4 (4.8%)	30 (44.8%) 13 (19.4%) 20 (29.9%) 4 (6%)	7 (43.8%) 1 (6.3%) 8 (50%) − (0%)	0.28
Restrictive TAPVC *	69 (83.1%)	57 (85.1%)	12 (75%)	0.265
Time from admission to operation (days)	8.4 (16.2; 0–99)	6.9 (15; 0–99)	14.8 (20; 0–61)	0.162

* definition in the text; TAPVC—total anomalous pulmonary venous connection; SD—standard deviation.

**Table 2:** Surgical and postoperative data of children with TAPVC.

Feature	Total (*n* = 83); Mean (SD; Range) or Number (%)	Normal Heart Physiology (*n* = 67); Mean (SD; Range) or Number (%)	Single Ventricle Physiology (*n* = 16); Mean (SD; Range) or Number (%)	*p*-Value
CPB time (min.)	152 (68.3; 45–441)	147 (70; 45–441)	176 (57; 74–305)	0.016
AoX time (min.)	53.5 (20.4; 21–118)	56 (20.4; 21–118)	43 (16.7; 27–78)	0.015
Use of DHCA	73 (88%)	57 (85.1%)	16 (100%)	0.102
DHCA time (min.)	47.9 (18.2; 20–104)	52 (18.1; 20–104)	33 (7.2; 22–52)	0.004
Delayed sternal closure	36 (43.4%)	26 (38.8%)	10 (6.3%)	0.086
Complications (all):	50 (60.3%)	34 (50.8%)	16 (100%)	<0.001
- ventilation over 7 days	45 (54.2%)	29 (43.3%)	16 (100%)	<0.001
- temporary kidney insufficiency requiring dialysis	31 (37.4%)	19 (28.4%)	12 (75%)	0.001
- postoperative arrhythmia	13 (15.7%)	8 (11.9%)	5 (31.3%)	0.056
- major infections	10 (12.1%)	6 (9%)	4 (25%)	0.095
Postoperative mechanical ventilation time (hours)	291 (443.8; 1–2750)	180 (213.6; 1–1080)	757 (767.7; 4–2750)	<0.001
ICU stay (days)	17 (29.5; 0–205)	9 (10.6; 0–49)	49 (54.3; 0–205)	<0.001
Postoperative hospital stay (days)	26 (29; 0–206)	19 (11.9; 1–62)	54 (52.8; 0–206)	<0.001

TAPVC—total anomalous pulmonary venous connection; SD—standard deviation; CPB—cardiopulmonary bypass; AoX—aortic cross-clamp; DHCA—deep hypothermic circulatory arrest; ICU—intensive care unit.

**Table 3:** Cox proportional hazards model for the patients, after TAPVC correction associated with overall mortality.

	Univariate Analysis			Multivariate Analysis		
Covariate	HR	95%CI	*p*-Value	HR	95%CI	*p*-Value
Preoperative features						
Age (days)	0.996	0.952–1.01	0.531			
Newborn	1.975	0.562–6.939	0.289			
Gender	0.598	0.192–1.857	0.374			
Restrictive TAPVC *	1.067	0.304–3.745	0.92			
TAPVC subtype	0.742	0.442–1.247	0.261			
Supracardiac (I) vs. others (II, III, and IV) subtype of TAPVC	2.568	0.932–7.076	0.068	3.61	1.258–10.356	0.017
Intracardiac (II) vs. others (I, III, and IV) subtype of TAPVC	0.037	0–9.201	0.241			
Infracardiac (III) vs. others (I, II, and IV) subtype of TAPVC	0.8	0.278–2.303	0.679			
Mixed (IV) vs. others (I, II, and II subtype) of TAPVC	0.965	0.127–7.308	0.972			
Single ventricle physiology	9.125	3.326–25.04	<0.001	9.126	3.259–25.553	<0.001
Weight (kg)	0.643	0.324–1.277	0.207			
Weight below 3 kg	2.242	0.834–6.025	0.109	2.064	0.737–5.783	0.168
Intra- and postoperative features						
Time from admission to operation (days)	1.015	0.996–1.034	0.114	1.657	1.273–2.156	<0.001
Postoperative mechanical ventilation time (hours)	1.001	1–1.002	0.001	0.999	0.998–1.001	0.313
ICU stay (days)	1.013	1.005–1.021	0.001	1.624	1.256–2.1	<0.001
Postoperative hospital stay (days)	1.014	1.006–1.022	0.001	0.608	0.468–0.791	<0.001
Postoperative complications (overall)	63.366	1.112–3612.041	0.044	0.122	0–146+	0.99
Postoperative mechanical ventilation over seven days	73.552	1.297–4141.414	0.037	14020.79	0–146+	0.955
Temporary kidney insufficiency requiring dialysis	34.693	4.565–263.661	0.001	38.376	2.976–494.831	0.005
Postoperative arrythmia	2.964	1.016–8.645	0.047	2.534	0.445–14.425	0.295
Major infections	0.607	0.08–4.595	0.628			
Delayed sternum closure	1.538	0.575–4.114	0.392			
CPB time (min.)	1.007	1.001–1.012	0.013	1.003	0.994–1.011	0.544
AoX time (min.)	1.004	0.98–1.029	0.741			
Use of DHCA (min.)	1.853	0.244–14.045	0.551			
DHCA time (min.)	0.999	0.97–1.029	0.947			
Need of reoperation	2.944	0.667–13.007	0.154	5.906	0.74–47.110	0.094

* definition in the text; TAPVC—total anomalous pulmonary venous connection; CPB—cardiopulmonary bypass; AoX—aortic cross-clamp; DHCA—deep hypothermic circulatory arrest; ICU—intensive care unit.

## Data Availability

Data are available on request, due to restrictions, e.g., privacy or ethical.
